# Supplementary material for: Integrated Systems Pharmacology, Urinary Metabonomics, and Quantitative Real-Time PCR Analysis to Uncover Targets and Metabolic Pathways of the You-Gui Pill in Treating Kidney-Yang Deficiency Syndrome
Source: Int J Mol Sci. 2019 Jul 26;20(15):3655. doi: 10.3390/ijms20153655 (PMC6696241; doi:10.3390/ijms20153655)
Supplement: Supplementary file 1 [file ijms-20-03655-s001.zip › Supplementary File(s)/Supplementary Material (Fig. S1-S3).pdf]

Figure S1. The variation of weights of the rats among the three groups

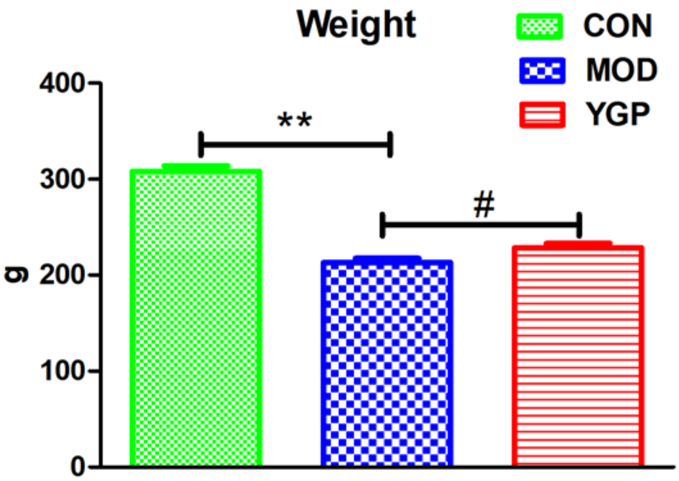

Note: $^{*}P < 0.05$, $^{**}P < 0.01$; $^{\#}P < 0.05$, $^{\#\#}P < 0.01$

Figure S2. Permutation test plots (200 permutations) from urine samples: (A) CON group *vs* MOD group; (B) MOD group *vs* YGP group

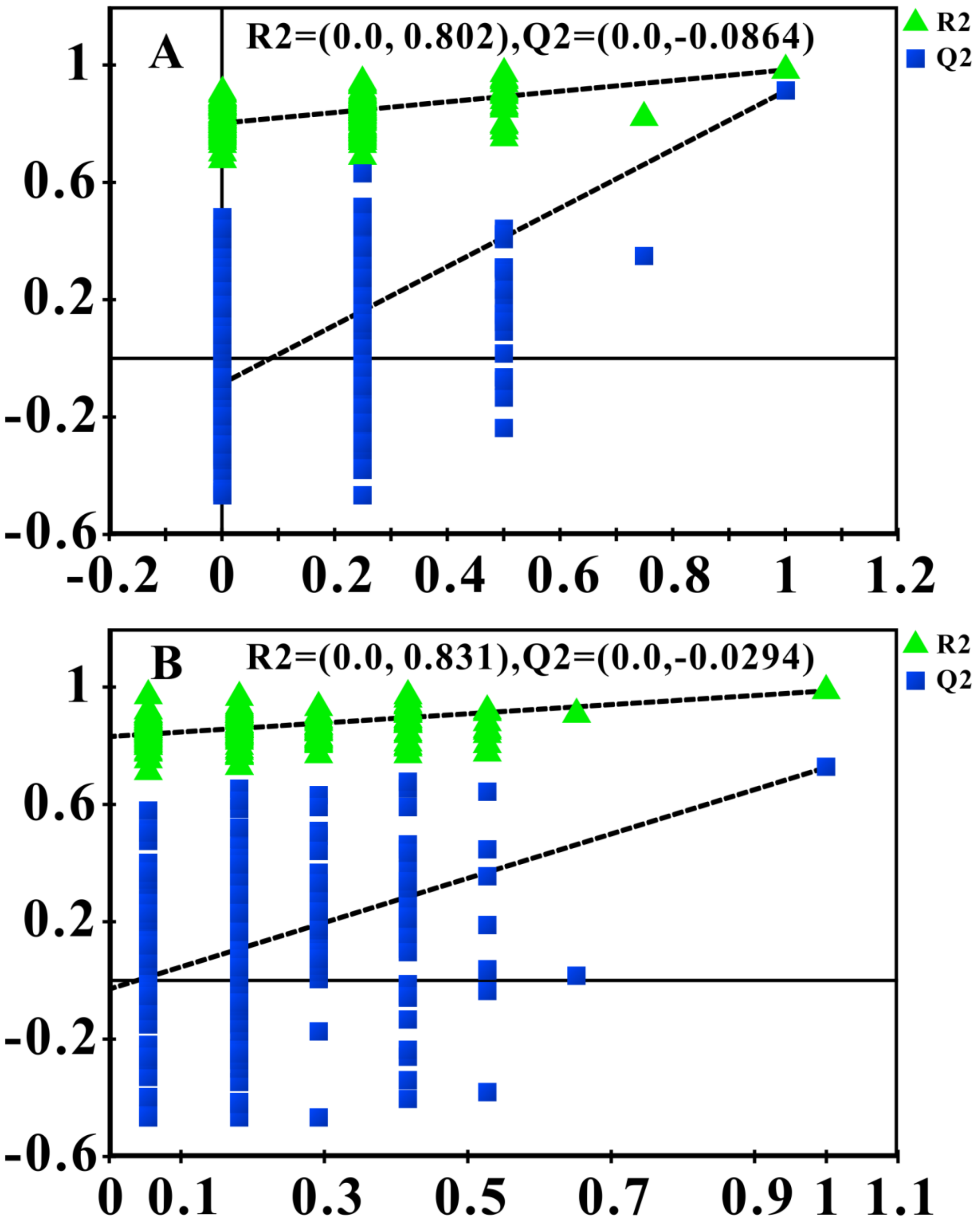

Figure S3. The 2D structure diagram of ten active compounds in YGP

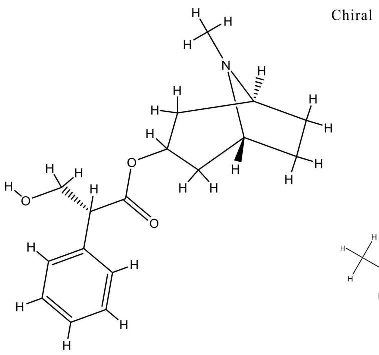

Atropine

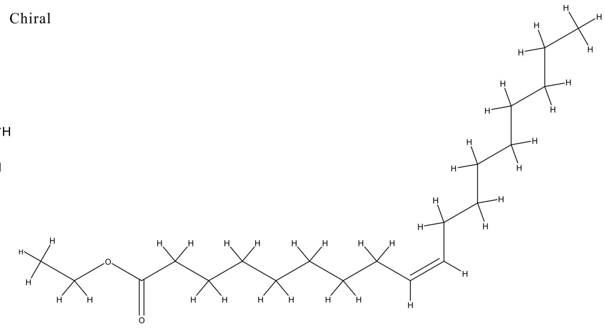

Ethyl oleate (NF)

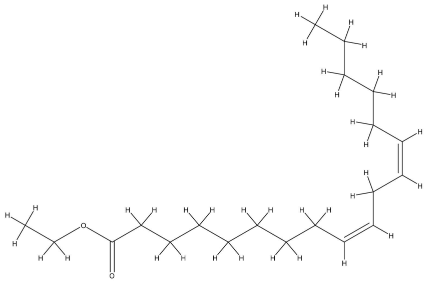

Mandenol

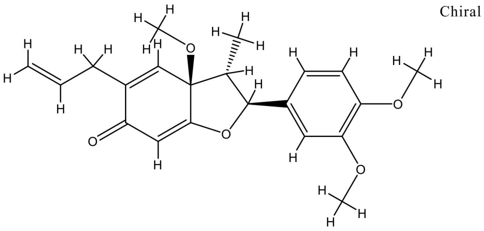

Kadsurenone

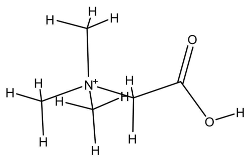

Betaine

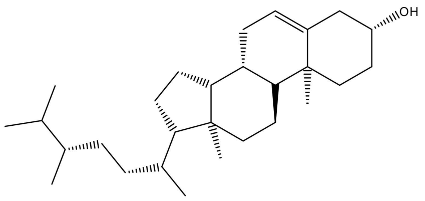

24-Methylcholest-5-enyl-3belta -O-glucopyranoside_qt

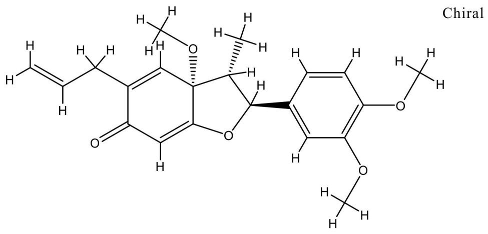

Denudatin B

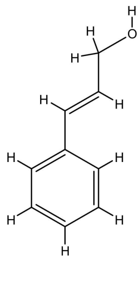

Styrone

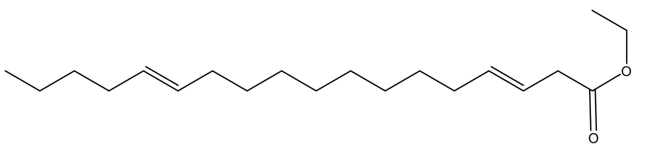

(E,E)-1-ethyl octadeca-3,13-dienoate

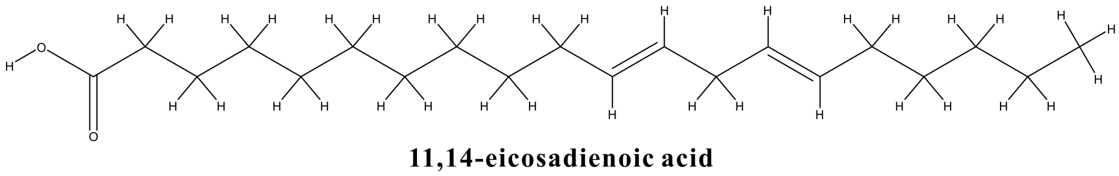

11,14-eicosadienoic acid
