# Supplementary material for: Integrated Systems Pharmacology, Urinary Metabonomics, and Quantitative Real-Time PCR Analysis to Uncover Targets and Metabolic Pathways of the You-Gui Pill in Treating Kidney-Yang Deficiency Syndrome
Source: Int J Mol Sci. 2019 Jul 26;20(15):3655. doi: 10.3390/ijms20153655 (PMC6696241; doi:10.3390/ijms20153655)
Supplement: Supplementary file 1 [file ijms-20-03655-s001.zip › Supplementary File(s)/Supplementary Material (Table S1-S2).pdf]

Supplementary Material

Table S1. Statistical analysis results of the main metabolite in urine

No.	Metabolites	Chemical shift (ppm)	VIP values in MOD *vs* CON	VIP values in YGP *vs* MOD
1	2-hydroxy isovalerate	0.82d, 0.95d	↓**(1.19893)	↑##(1.4592)
2	2-oxoisocaproate	0.93d	↓**(1.20244)	↑##(1.3372)
3	valine	0.98d, 1.03d, 2.29(m), 3.62(d)	↓*(2.3414)	↑##(1.16657, 1.9085)
4	3-hydroxybutyrate	1.20d	↓(1.27208)	↑#(1.80074)
5	lactate	1.33d, 4.11q	↓**(1.43589, 1.82228)	↑##(3.22914, 2.00244)
6	alanine	1.48d	↓**(1.45455)	↑##(1.58037)
7	acetate	1.91s	↓(3.21613)	↑#(6.38963)
8	NAG	2.04s	↓**(4.8771)	↑##(3.44076)
9	acetone	2.23s	↓(1.49277)	↑##(1.5611)
10	succinate	2.41s	↓**(2.44932)	↑##(3.71412)
11	α-ketoglutarate (α-KG)	2.45t, 3.01t	↓(2.50328, 1.3932)	↑##(3.03184, 3.66234)
12	citrate	2.56d, 2.72d	↓**(3.93786, 2.30092)	↑(1.63101, 2.51258)
13	dimethylamine (DMA)	2.71s	↓*(1.06588)	↑#(1.91873)
14	dimethylglycine (DMG)	2.92s	↓*(3.86941)	↑(1.81422)
15	creatinine	3.05s, 4.06s	↓*(5.46773, 4.18704)	↑(2.08887,1.68475)
16	TMAO	3.27s	↓**(4.25915)	↑(2.70055)
17	choline	3.20s	↓*(1.74248)	↑#(1.56094)
18	glycine	3.56s	↓(1.79166)	↑#(2.09787)
19	glycerol	3.64dd, 3.87dd	↓**(2.66159, 2.17052)	↑##(1.84109, 1.70949)
20	creatine	3.93s	↓**(2.04625)	↑##(1.63151)
21	α-glucose	5.24d, 3.70t, 3.40t, 3.83dd, 3.72dd, 3.85m	↓**(2.12986, 1.14185, 2.47749, 2.51527, 2.43068)	↑##(2.02001, 1.01783, 1.80521, 2.18719, 1.61842)

22	β-glucose	4.65d, 3.24dd, 3.47dd	↓**(1.18783, 1.60082,)	↑#(1.04652)
23	betaine	3.90s	↓**(3.31971)	↑##(2.16067)
24	hippurate	3.97d, 7.55t, 7.64t, 7.84d	↓**(1.43334, 1.80337)	↑(1.12264, 1.7032)
25	tryptophan	7.21t, 7.29t, 7.56d	↓**(1.11104, 1.94659)	↑##(1.83171)
26	phenylalanine	7.33m, 7.44m, 7.39(m), 3.17(dd), 3.30(dd), 3.99(dd)	↓*(1.16678, 1.78083)	↑##(1.24986, 1.45088)
27	formate	8.46s	↓(1.45119)	↑#(2.57806)

Note: *P* value was measured by one-way ANOVA with a Bonferroni correction, $^{*}P < 0.05$, $^{**}P < 0.01$; $^{\#}P < 0.05$, $^{\#\#}P < 0.01$. The chemical shift values of marked green corresponded to the VIP values in MOD *vs* CON group, while the chemical shift values of labeled red corresponded to the VIP values in YGP *vs* MOD group.

Table S2. Results of pathway analysis using MetaboAnalyst

No.	Pathway Name	Match Status	Raw p	-log(p)	Holm p	FDR	Impact	Details
1	Glycine, serine and threonine metabolism	5/32	1.40E-04	8.8722	0.011359	0.011359	0.3236	KEGG
2	Butanoate metabolism	3/20	0.0041543	5.4836	0.33235	0.090967	0	KEGG
3	Citrate cycle (TCA cycle)	3/20	0.0041543	5.4836	0.33235	0.090967	0.14721	KEGG
4	Aminoacyl-tRNA biosynthesis	5/67	0.0044922	5.4054	0.35039	0.090967	0	KEGG
5	Alanine, aspartate and glutamate metabolism	3/24	0.0070505	4.9547	0.54289	0.11422	0.06329	KEGG
6	Methane metabolism	2/9	0.0093998	4.6671	0.71438	0.1269	0	KEGG
7	Valine, leucine and isoleucine biosynthesis	2/11	0.014063	4.2642	1	0.16273	0.33333	KEGG
8	Glyoxylate and dicarboxylate metabolism	2/16	0.029123	3.5362	1	0.29487	0.40741	KEGG
9	Pyruvate metabolism	2/22	0.052676	2.9436	1	0.47408	0.05583	KEGG
10	Phenylalanine, tyrosine and tryptophan biosynthesis	1/4	0.066805	2.706	1	0.4792	0.5	KEGG
11	Glycolysis or Gluconeogenesis	2/26	0.071108	2.6435	1	0.4792	0.02862	KEGG
12	Galactose metabolism	2/26	0.071108	2.6435	1	0.4792	0.03644	KEGG
13	Synthesis and degradation of ketone bodies	1/5	0.082826	2.491	1	0.4792	0	KEGG
14	D-Glutamine and D-glutamate metabolism	1/5	0.082826	2.491	1	0.4792	0	KEGG
15	Cyanoamino acid metabolism	1/6	0.098582	2.3169	1	0.53234	0	KEGG
16	Valine, leucine and isoleucine degradation	2/38	0.136	1.9951	1	0.6494	0.0119	KEGG
17	Nitrogen metabolism	1/9	0.14431	1.9358	1	0.6494	0	KEGG
18	Phenylalanine metabolism	1/9	0.14431	1.9358	1	0.6494	0.40741	KEGG
19	Selenoamino acid metabolism	1/15	0.22919	1.4732	1	0.92822	0	KEGG
20	Pantothenate and CoA biosynthesis	1/15	0.22919	1.4732	1	0.92822	0	KEGG

21	Glycerolipid metabolism	1/18	0.26854	1.3147	1	1	0.28098	KEGG
22	Propanoate metabolism	1/20	0.2937	1.2252	1	1	0	KEGG
23	Starch and sucrose metabolism	1/23	0.32989	1.109	1	1	0.03778	KEGG
24	Glutathione metabolism	1/26	0.36429	1.0098	1	1	0.00573	KEGG
25	Porphyrin and chlorophyll metabolism	1/27	0.37538	0.97981	1	1	0	KEGG
26	Glycerophospholipid metabolism	1/30	0.40754	0.8976	1	1	0.02315	KEGG
27	Tryptophan metabolism	1/41	0.51243	0.66859	1	1	0.15684	KEGG
28	Arginine and proline metabolism	1/44	0.53779	0.62029	1	1	0.01198	KEGG
29	Primary bile acid biosynthesis	1/46	0.55399	0.59061	1	1	0.02976	KEGG

Note: The "Match Status" represents the ratio of actual matched number from the user uploaded data to the number of compounds in the pathway; the *p* values from the pathway enrichment analysis and pathway impact values (from 0 to 1) from the pathway topology analysis.
